# Is Sustainability Part of the Drill? Examining Knowledge and Awareness Among Dental Students in Bucharest, Romania

**DOI:** 10.3390/dj13030114

**Published:** 2025-03-05

**Authors:** Ana Maria Cristina Țâncu, Marina Imre, Laura Iosif, Silviu Mirel Pițuru, Mihaela Pantea, Ruxandra Sfeatcu, Radu Ilinca, Dana Cristina Bodnar, Andreea Cristiana Didilescu

**Affiliations:** 1 Department of Prosthodontics, Faculty of Dentistry, “Carol Davila” University of Medicine and Pharmacy, 041292 Bucharest, Romania; anamaria.tancu@umfcd.ro (A.M.C.Ț.); mihaela.pantea@umfcd.ro (M.P.); 2 Department of Organization, Professional Legislation and Management of the Dental Office, Faculty of Dentistry, “Carol Davila” University of Medicine and Pharmacy, 041292 Bucharest, Romania; silviu.pituru@umfcd.ro; 3 Department of Oral Health and Community Dentistry, Faculty of Dentistry, “Carol Davila” University of Medicine and Pharmacy, 041292 Bucharest, Romania; ruxandra.sfeatcu@umfcd.ro; 4 Department of Medical Biostatistics and Informatics, Faculty of Dentistry, “Carol Davila” University of Medicine and Pharmacy, 041292 Bucharest, Romania; radu.ilinca@umfcd.ro; 5 Department of Operative Dentistry, “Carol Davila” University of Medicine and Pharmacy, 041292 Bucharest, Romania; dana.bodnar@umfcd.ro; 6 Department of Embryology and Microbiology, Faculty of Dentistry, “Carol Davila” University of Medicine, 010221 Bucharest, Romania; andreea.didilescu@umfcd.ro

**Keywords:** environmental sustainability, development, dentistry, dental students, faculty, curriculum, elective course, dental practice, carbon footprint

## Abstract

**Background.** Despite dentistry’s alarmingly high energy use, plastic waste, and travel emissions, research on Romanian dental students’ sustainability awareness is absent. This study aimed to assess their knowledge of the environmental impact of dental materials and practices, hypothesizing that early exposure to sustainability education would benefit preclinical students most. **Materials and Methods.** A cross-sectional survey using a form questionnaire with 15 items was conducted on 1800 dental students at Carol Davila University of Medicine and Pharmacy, Bucharest, Romania, for one week in March 2022. The questionnaire, consisting of socio-demographics, students’ perspectives on sustainability in dentistry, and personal sustainability, was analyzed using SPSS 26. Data analysis included the Shapiro–Wilk test for normality, Fisher’s exact test for categorical variables, the Mann–Whitney U test for non-parametric quantitative comparisons, and Z-tests with Bonferroni correction for contingency tables. **Results.** A response rate of 26.06% was achieved, with 469 participants. The majority (51.1%), particularly males (66.1%), perceived sustainability as promoting durability. The most common definition of sustainability (33.8%) was related to environmental protection, with significantly higher agreement among female students (39.4%) (*p* = 0.001). While 49.3% of participants identified single-use plastics in patient care as having the greatest environmental impact in dental practices, 39.2% of female students, primarily from clinical study years (50%), ranked patient paperwork and records as the most significant factor (*p* = 0.031). The highest-carbon-footprint dental procedures were considered to be amalgam and composite fillings (50.7%), with clinical year students indicating this as the most relevant issue (62.8% vs. 47.7%) (*p* = 0.011). Students aged 25–30 were more actively engaged in sustainability initiatives compared to the younger group (*p* = 0.005), while all students over 30 identified scaling and polishing as the most impactful procedure (*p* < 0.001). A majority of students supported future university sustainability initiatives (62.7%) and an elective course on sustainability in dentistry (65%). Female students showed significantly greater interest than male students in both initiatives (66.3% vs. 52.7%, *p* = 0.003 and 70.8% vs. 49.6%, *p* < 0.001, respectively). **Conclusions.** Greater awareness of sustainability was found in preclinical-year dental students and among female students, with knowledge gaps in clinical-year students, particularly regarding the environmental impact of dental practices and materials. Introducing sustainability courses could better prepare future dentists for sustainable practices in dentistry. Research collaborations and curriculum reforms to further promote sustainability would also be beneficial.

## 1. Introduction

In 1987, the United Nations Brundtland Commission defined sustainability as “meeting the needs of the present without compromising the ability of future generations to meet their own needs” [1]. Today, this concept as described by the latest report [2] states that sustainability requires reducing environmental impact while supporting systems that allow for adaptation and mitigation of risks, particularly in the context of climate resilience, highlighting the balance between an individual’s quality of life and environmental resilience [3]. Also described as an umbrella term [4], it encompasses concerns about climate change, carbon emissions, and plastic pollution [5,6] that impact both the environment and the individual, as well as broader societal well-being [7]. That is the reason why this multi-dimensional concern has gained substantial global traction by 2023, with over 130 countries adopting sustainability strategies in line with the United Nations (UN) Sustainable Development Goals (SDGs) [8,9].

As environmental sustainability and human health are deeply interwoven, two relatively recent concepts—sustainable healthcare and green medicine—have become focal points for global organizations like the World Health Organization (WHO) [10] and the World Health Assembly (WHA) [11], which focus on aligning healthcare practices with environmental stewardship, social equity, and long-term sustainability. Paradoxically, meeting the population’s health needs negatively affects the environment, as healthcare is a major source of pollution due to high CO_2_ emissions and waste [12,13,14].

Notably, dentistry is recognized as one of the most unsustainable medical specialties due to three key mechanisms of environmental pollution: significant energy consumption, extensive use of single-use plastics, and considerable kilometers traveled [15,16] by doctors, staff, and patients, to and from the dental practice [17]. With no exceptions, all dental specialties contribute to waste generation and environmental unsustainability, although the degree of their impact varies. Among all, prosthodontics can be regarded as one of the most resource-demanding fields and a high contributor to carbon emissions due the substantial energy required for the manufacturing process [18], as well as for the use of various high-impact chemicals and materials, including polymers, ceramics, metals, gypsum, and wax, which are frequently used in large quantities and disposed of after a single use [19]. Other dental specialties with significant environmental impact, such as orthodontics [20], restorative dentistry [21,22,23,24,25], and oral and maxillofacial surgery [26,27], follow prosthodontics as the main contributors to resource consumption, waste generation, and carbon emissions.

As humanity has reached almost finite resources [28], it is unsurprising that in the past two decades, significant steps have been taken to mitigate the environmental impact of traditional dentistry. Dentistry is progressively transforming into an eco-friendly, green specialty [29,30], emphasizing the role of social stakeholders in sustainable oral healthcare development. This shift relies on key pillars, including oral health professionals and associations [25,31], which develop and implement guidelines for eco-friendly materials like recyclable metals, plant-based polymers, and non-toxic composites [32]. Sustainable measures such as energy-efficient equipment, water-saving sterilizers, and the reduction of single-use plastics are essential in dental practices [29]. Furthermore, the integration of advanced digital technologies like CAD/CAM systems, 3D printing, and AI tools [33,34] plays a critical role in reducing waste, packaging, and transportation needs between laboratories and clinics [35].

The conversion of the conventional dental patient into a sustainable counterpart through awareness constitutes the second social pillar in the development process of green dentistry. In this regard, the dental patient may contribute to immediate benefits in green dentistry through the adoption of sustainable practices such as minimizing single-use dental items, embracing smart treatment combinations and shared family appointments [36], reducing energy consumption by opting for strategically scheduled dental visits [37], using eco-conscious oral hygiene products, or promoting green dentistry practices.

Nevertheless, the most compelling driving pillar toward sustainable dentistry, with enduring benefits for future generations, is undoubtedly the incorporation of environmental concepts into dental education, specifically through the embedding of sustainability principles into university curricula [38]. While specific statistics on the exact number of universities worldwide that incorporate sustainability into their curricula are not readily available, amid growing concerns about the enormous current levels of resource consumption, energy use, and waste production globally, the trend toward integrating sustainability into higher education is increasing significantly [39].

The stringent need to embed environmental sustainability within dental higher education has also taken root in Romania in recent years [40], driven one the one side by the necessity of aligning with European standards [41,42], and one the other side by the global awareness of the critical need to secure the survival of future generations due to diminishing resources. Currently, at the time of writing this manuscript, the strategic development plan of the Carol Davila University of Medicine and Pharmacy for the period 2021–2029 [43] places significantly greater emphasis on environmental sustainability, a shift that demonstrates the rapid awareness of the need to prioritize sustainability measures within our academic community.

The perception of dental students regarding sustainability measures in dental practice, as well as in their personal lives, has been a widely discussed topic in international scientific literature [44,45,46,47] and, at the same time, an efficient starting point for incorporating specific curriculum subjects and standalone environmental sustainability courses into undergraduate dentistry education [31,48].

Despite prior research on private dental entrepreneurship in Romania [15,48,49], no studies have examined dental students’ perceptions, knowledge, and awareness of environmental sustainability. Moreover, sustainability is absent from Romanian dental curricula. Addressing this gap, this study aimed to assess students’ knowledge of the environmental impact of dental materials and practices, hypothesizing that older students, females, and those in senior years would show greater awareness. A secondary objective was to evaluate the need for an elective course on sustainability in dental education.

## 2. Materials and Methods

### 2.1. Study Design and Sampling Procedures

A cross-sectional survey was conducted on students from the Faculty of Dentistry at Carol Davila University of Medicine and Pharmacy in Bucharest, Romania. All 1800 dental students enrolled in the Dentistry program for the 2022–2023 academic year were deemed eligible for participation and were invited via institutional email to anonymously complete a Type form questionnaire.

### 2.2. Sample Size Calculation

The sample size for this study was determined using GPower 3.1.9.7 software (University of Kiel, Kiel, Germany), based on a medium effect size (φ = 0.3), a significance level (α) of 0.05, and a statistical power of 0.8. The analysis considered contingency tables with a structure of 2 × 2, and potentially 6 × 2, to maximize the study’s statistical power. The minimum required sample size was calculated to be 143 participants for a statistical power of 0.8, or 297 participants for an optimal power of 0.99.

### 2.3. Data Collection and Ethical Consideration

The pilot questionnaire was reviewed by the research team to assess the precision of the wording, and the questions were carefully examined to ensure they comprehensively addressed all the study objectives. The final version of the questionnaire in English, along with all required documentation, received ethical approval from the Ethics Commission of Scientific Research at Carol Davila University of Medicine and Pharmacy in Bucharest, Romania. The approval was granted under reference number 6118/08.03.2022. On 11 March 2022, the questionnaire was distributed via institutional email by the lead student of each university year. Participants were given a one-week window to access and complete the questionnaire. An introductory statement accompanied the questionnaire, detailing the study’s objectives. It was clearly stated that participation was voluntary, no incentives were provided, and anonymity was guaranteed. Students were informed that they could skip any question they did not wish to answer without affecting their participation or the validation of their responses to other questions. Participants were also informed of their right to decline participation at any point. Completion and submission of the questionnaire were regarded as implicit consent to participate in the study.

### 2.4. Survey Instrument

A Type form questionnaire with 15 items was developed by the authors of this study in collaboration with Bemari.co.uk, a consultancy specializing in sustainability and procurement. Bemari.co.uk provides advisory services to both public and private sector organizations, focusing on sustainable operational practices, resource efficiency, environmental management, and climate policy. Their expertise in sustainability consulting made them an ideal partner for ensuring that the questionnaire addressed key sustainability issues relevant to the study. Following in-depth discussions with the external expert, the initial version of the questionnaire was created in Romanian and reviewed and approved by all study authors. It was then back-translated into English by two bilingual individuals fluent in both English and Romanian. The English version was subsequently evaluated by a group of 10 doctoral dental students, who provided assessments on the clarity and comprehensibility of the items. Based on their assessments, the final version was used in the current study.

The Type form was chosen as the survey instrument for this study due to its user-friendly, interactive design, which has been shown to increase participant engagement [50], especially in younger demographics [51], such as university students. The Type form’s intuitive interface allows for a conversational flow that encourages more thoughtful and complete responses, making it particularly effective for both open-ended and closed-ended questions. The questionnaire combined mandatory open-ended questions, which provided qualitative insights, with closed-ended questions for quantitative analysis. The open-ended responses were coded into predefined categories for subsequent statistical analysis, and these codes will be detailed below, along with the description of the questionnaire. The complete questionnaire can be found by accessing Supplementary File Table S1. To maintain data quality, duplicate responses were automatically identified and eliminated through Type form’s built-in tools. These tools allowed for the detection of multiple submissions from the same participant based on unique identifiers, such as email addresses or IP addresses. Redundant responses were then removed from the dataset, ensuring that only unique contributions were included in the final analysis.

The first section, titled “About You”, aimed to collect personal data from the respondents, including three closed-ended questions (Q1–Q3) on their year of study, age group, and gender. Part two, titled “Your perspectives on sustainability issues in dentistry”, consisted of 9 questions (Q4–Q12), including 4 open-ended questions (Q4, Q6, Q10, and Q11), 3 multiple-choice questions (Q7, Q8, Q9), 1 single-choice question (Q12), and 1 score-based question rated from 1 (not important) to 10 (essential) (Q5). The coding for the open-ended responses to Q4 was as follows: 1. Ecological; 2. Durable; 3. Economic; 4. Other. The coding for the open-ended responses to Q6 was represented as follows: 1. For environmental protection; 2. For promoting the patient’s health; 3. For long-lasting procedures; 4. For promoting material quality; 5. For increasing the quality–price ratio; 6. For self-evolution in dental practice. The responses to Q10 were coded as follows: 1. Dental office modernization; 2. Reducing waste and using eco-friendly materials; 3. Acquiring more durable materials; 4. Self-education about environmentally friendly practices. The last open-ended question in this section, Q11, was coded with the following options: 1. Yes; 2. No; 3. Undecided. The final section, “Your personal sustainability overview”, included 2 multiple-choice questions (Q13, Q15), and 1 single-choice question (Q14), all of which, along with the entire questionnaire, can be accessed by opening the Supplementary File Table S1.

### 2.5. Data Analysis

Data were analyzed using IBM SPSS Statistics, version 26 (Armonk, NY, USA: IBM Corp.), and illustrated using Microsoft Office Excel/Word 2016 (Microsoft Corporation, Redmont, WA, USA). Quantitative variables were assessed for normal distribution using the Shapiro–Wilk test and presented as means with standard deviations or medians with interquartile ranges, depending on distribution. Qualitative variables were reported as counts or percentages, and differences between groups were evaluated using Fisher’s exact test. For quantitative independent variables with non-parametric distribution, Mann–Whitney U tests were used to compare groups. Z-tests with Bonferroni correction were applied to further detail the results obtained from contingency tables.

## 3. Results

In this study, 469 students participated effectively, ensuring a response rate of 26.06%. Both the minimum required sample size of 143 participants for a level of statistical power of 0.8 and the optimal sample size of 297 participants for a level of statistical power of 0.99 were exceeded. Thus, the sample size ensured the reliability and validity of the statistical analysis, providing strong evidence for the study’s findings. Of the 469 undergraduate dental students, 342 were females (72.9%). Demographic data, such as age, gender, and study year, can be found by accessing Supplementary File Table S2. Most of the dental students were in the preclinical stage (first to third study year) (79.9%), while 20.1% of them were in the clinical stage of the program (fourth to sixth study year). Most of the students were in the 18–24 age group (93.4%).

The students’ distribution based on the study’s answers were as follows. Questions 4, 6, 10, and 11 were open-ended, and the answers were manually categorized. Questions 7, 8, 9, 13, and 15 were multiple-choice, while question 5 was a score-based question ranked from 1 point (not important) to 10 points (essential). The results, which can be accessed in Supplementary Table S3, show the following:

- In terms of sustainability, most students considered it a quality that promotes durability (51.1%) or environmentally friendly services (28.9%);
- The importance of sustainability practices was considered very high (median = 9 points, IQR = 8–10 points);
- Most of the students considered that sustainability is relevant in the dental profession for environmental protection (33.8%) or for self-evolution in the dental practice (32%);
- The activities most associated with the highest adverse environmental impact were the use of single-use cups (49.3%) and use of specialist materials (46.3%);
- The use of modern techniques and technologies was the most frequently chosen aspect that could affect improving the environment (33.9%);
- The top three dental procedures with the most significant carbon footprint were considered to be amalgam and composite fillings (50.7%), radiographs (36.9%), and metal dentures (34.1%);
- Most of the students considered that the most relevant opportunities for the dental profession to improve its environmental impact were reducing waste while using eco-friendly materials (38.4%) or the modernization of the dental office (29.5%);
- 62.7% of the students said they would want to see the university undertake initiatives to promote sustainability;
- 65% of the students said they would want to attend a possible elective course on sustainability in dentistry;
- 58.6% of the students considered that they were worried about the environment and had made some lifestyle changes but were unsure about what else to do;
- Only 14.7% of the students were involved in sustainability initiatives or organizations;
- The students’ most frequent lifestyle choices to promote sustainability were turning off lights (69.7%) and using only public transport (53.3%).

In order to test the study hypothesis, the distribution of students according to age group and other items answered in the survey was analyzed, as indicated by Table 1. Key findings from this were as follows:

- Students aged over 30 years responded significantly more frequently that scale and polish is among top three dental procedures with the largest carbon footprint than students aged between 18 and 24 years or 25 and 30 years (100% vs. 20.1%/38.5%) (*p* < 0.001);
- Students aged between 25 and 30 years responded significantly more frequently that they were involved in sustainability initiatives than students aged between 18 and 24 years (34.6% vs. 13.2%) (*p* = 0.005);
- Students between 18 and 24 years old responded significantly more frequently that they used only public transport than students between 25 and 30 years old (55% vs. 30.8%) (*p* = 0.014).

Also, the study hypothesis referred to the influence of gender on both knowledge and awareness regarding professional and personal sustainability. Data from Table 2 show the distribution of students according to gender and other items answered in the survey:

- In terms of the sustainability concept, female students considered more frequently that sustainability is a quality that promotes environmentally friendly services (33.7% vs. 16.1%), while male students considered more frequently that sustainability is a quality that promotes durability (66.1% vs. 45.5%) (*p* = 0.001);
- In terms of sustainability relevance for dental profession, female students considered more frequently that sustainability is relevant for environmental protection (39.4% vs. 17.9%), while male students considered more frequently that sustainability is relevant for promoting long-lasting procedures (14.3% vs. 7.1%) or self-evolution in the dental practice (45.2% vs. 27.4%) (*p* = 0.001);
- Female students were more frequently interested in university initiatives (66.3% vs. 52.7%) (*p* = 0.003) and in optional courses about sustainability (70.8% vs. 49.6%) (*p* < 0.001) than male students;
- Female students considered more frequently that patient paperwork and records (39.2% vs. 28.3%) (*p* = 0.031) and dental office lighting and energy (26.9% vs. 15.7%) (*p* = 0.014) had the highest adverse environmental impact than male students;
- Female students responded more frequently that they used organic food (30.4% vs. 20.5%) (*p* = 0.037), recycled waste (55.6% vs. 44.1%) (*p* = 0.029), and bought less (46.2% vs. 29.9%) (*p* = 0.002) than male students.

Data from Table 3 show the distribution of students according to study year and other items answered in the survey, as follows:

- In terms of the sustainability concept, students from study years IV to VI considered more frequently that the meaning of the term “sustainability” was ecological (40.4% vs. 25.8%) (*p* = 0.022);
- Students from study years I–III (median = 9 points, IQR = 8–10 points) had significantly higher values for the sustainability awareness importance score than the students from study years IV–VI (median = 9 points, IQR = 7–10 points) (*p* = 0.028);
- Students from study years IV–VI considered more frequently that patient paperwork and records (50% vs. 32.8%) (*p* = 0.003) and amalgam and composite fillings (62.8% vs. 47.7%) (*p* = 0.011) had the highest adverse environmental impact than students from study years I–III;
- Students from study years I–III considered more frequently that endodontic treatments had the highest adverse environmental impact than students from study years IV–VI (19.2% vs. 9.6%) (*p* = 0.032) or did not know which treatments had the highest adverse environmental impact (31.2% vs. 20.2%) (*p* = 0.042);
- Students from study years IV–VI responded significantly more frequently that they were involved in sustainability initiatives than students from study years I–III (22.3% vs. 12.8%) (*p* = 0.023).

## 4. Discussion

In recent years, scarce scientific research has been conducted in dental faculties worldwide focusing on the level of knowledge and awareness among dental students regarding sustainability in dental practice. Most studies on dental students’ knowledge of environmental sustainability have been conducted in Europe and North America. Sustainability in dental education, initially prominent in Western countries, is gradually gaining traction in Eastern regions, which are increasingly recognizing the need to modernize their dental education programs to include sustainable practices. As sustainability awareness continues to grow, these efforts, although still in the early stages, reflect a growing commitment to eco-conscious practices.

Our survey achieved a higher-than-anticipated participation rate, given the type of questionnaire used [52] in comparison to the typical response rates for online surveys in higher education [53] or to other research with a similar target population and topic [53,54]. This indicates a significant level of interest in environmental sustainability among dental students at the Faculty of Dentistry, Carol Davila University of Medicine and Pharmacy, Bucharest. On the other hand, the response rate may appear moderate relative to other studies [55,56] and should therefore be interpreted with caution, given the variables affecting participation rates across different methodologies and contexts. For instance, Spaveras and Antoniado [55], utilizing a similar cross-sectional methodology within the Faculty of Dentistry at the National and Kapodistrian University of Athens (a single dental institution comparable to our study), achieved a higher participation rate of 39.2%. The higher participation rate can be attributed to the extended data collection period of six months, from April to October 2020, underscoring the significant role of longer accessibility in improving response rates. Supporting this observation are the findings of Haque et al. [46] from a year-long study targeting all undergraduate dental students across public and private dental colleges in Saudi Arabia, which achieved a significantly higher response rate of 64.7%. By contrast, our findings suggest that shorter collection periods, when well-promoted, can still yield valuable insights, albeit at a slightly reduced rate. The predominance of female participants and the overrepresentation of preclinical students from years I to III in our study appear to reflect characteristics of the respondent group rather than a specific bias in participation. This observation aligns with findings from a similar study conducted in 2021 on the same target population at the Faculty of Dentistry, Carol Davila University of Medicine and Pharmacy [56] and suggests that the demographic composition of our study participants is representative of the broader student population within the faculty.

Awareness among students regarding unsustainable dentistry was tested by assessing their perception of the profession’s responsibility for environmentally sustainable practices due to the fifth question. In addition to this, preclinical students from study years I–III demonstrated significantly higher awareness values regarding sustainability in dentistry compared to their clinical counterparts. This trend suggests that students in the early stages of their education may have a more idealistic or theoretical understanding of sustainability, whereas those in the clinical years may focus more on practical aspects of their professional training. Even so, most of our respondents allocated a very high level of importance to sustainable dental practices, with a median score of 9 out of 10. This finding aligns with the results of a study by Nassar et al. [47] where a high or very high agreement on the importance of sustainable dental practices of 86% of dental students in the United Arab Emirates (UAE) was reported. In our study, similar to their counterparts in the UAE, dental students demonstrated awareness of the growing responsibility of the profession in addressing an increasingly environmentally challenged world. Moreover, our study further indicates that female students exhibited greater knowledge of the relevance of sustainability for environmental protection.

The sixth question assessed students’ knowledge about the relevance of sustainability in the dental profession. From the multiple-choice response options, most students correctly correlated sustainability with environmental protection, highlighting a solid understanding of sustainability’s core principle, that is, minimizing environmental impact. However, these multiple-choice response options also allowed students to recognize sustainability not only as an environmental concept, but as one with broader implications for the dental profession. Responses such as promoting the patient’s health, ensuring long-lasting procedures, and enhancing material quality reflect an understanding of how sustainability contributes to improved patient care, procedural durability, and resource efficiency. This capacity to identify sustainability’s multi-dimensional relevance was the focus of this question, based on the premise that multiple-choice answers facilitate the testing of multi-dimensional knowledge [57].

Nearly half of the Romanian dental students explicitly recognized in their responses to the seventh question that the most significant environmental impact of dental activities stems from the use of single-use plastics (SUP), specialized materials, and dental equipment. These findings align with those reported by Durnall et al. [58], who observed a similar focus among UK dental students, particularly on the environmental concerns associated with SUP and personal protective equipment. Notably, such items are not always essential for cross-infection control purposes [59]. While the increasing use of plastics in dentistry has raised awareness about its environmental impact, it also underscores the need for more sustainable practices, especially as the dental sector continues to rely heavily on single-use items for infection control and patient safety. Acknowledging the alarming data among our students can be interpreted as a positive step forward, especially given the unprecedented explosion in single-use plastics in dental practice during the COVID-19 pandemic, and even the current post-pandemic era [47,60]. The widespread use of a myriad of SUP materials, including vinyl acrylics, polystyrene, epoxies, polycarbonates, polyethylene, polyvinyl acetate, polysulfides, polysilicon, polyethers, and acrylates [61] for items such as disposable tools and packaging materials has significantly contributed to environmental damage, as evidenced by the record-level data showing a 4.8% rebound in global CO_2_ emissions in 2021 [62]. Surprisingly, however, the group that did not identify the category of dentistry practices as the primary environmental threat were the clinical-year students. The lack of knowledge regarding the real impact of dental practices may result from the practical focus of the clinical years, which inadvertently diverts attention from sustainability practices, especially if they are not integrated into clinical training.

In the ranking of activities with potential to reduce environmental impact (the eighth question), the scientific literature places important attention on the use of modern digital technologies [18] or of modern biodegradable or eco-friendly dental materials. The latter include even materials with biodegradable properties used for over a decade, such as Resilon, a resin-based thermoplastic composite for root canal fillings [63]; recent adhesive formulations reinforced with nanoparticles [64]; green dental filling materials such as Zinc Oxide nanoparticles, chitosan nanoparticles, and glass ionomer mixtures [65]; or revolutionary biodegradable polymers for periodontal and endodontic coating purposes [66,67]. However, maximal environmental benefits are attributed to less specific activities, such as those that reduce the amount of resources utilized—the use of plastic medical disposals, water and electricity consumption—[68]. Nevertheless, given that most of our respondents were from preclinical years, the focus on modern dental materials (22%) and modern techniques and technologies (33.9%) was somewhat surprising. Preclinical students may view technology and modern materials as the future of green dentistry, where innovations tend to capture attention more than traditional or fundamental sustainability practices like reducing plastic waste or conserving water and electricity. This enthusiasm for technological advancement in the context of pro-environmental activities by students should be approached with caution, guiding them toward a more balanced perspective on the multi-faceted nature of sustainability in dentistry.

Regarding dental procedures with the highest carbon footprint, scientific reports have identified the top-ranking activities as examinations, followed by scaling and polishing, and radiographs, with amalgam and composite fillings occupying fourth place. From the responses to the ninth question in the questionnaire, the dental students in our study ranked the top three dental procedures with the highest carbon footprint as follows: amalgam and composite fillings (50.7%), radiographs (36.9%), and metal dentures (34.1%), with dental examinations placed fourth (29%). The observed discrepancies and similarities in the students’ perceptions of carbon footprint impacts reflect moderate gaps in their understanding, as procedures ranked first in the report were placed fourth by the students, while those ranked fourth rose to first place in their responses. Thus, students may underestimate the environmental impact of seemingly simple activities like examinations due to their less obvious resource consumption (e.g., single-use disposables, energy use for lighting and sterilization, water consumption), an observation that both confirms and complements the findings from the previous question. Another discrepancy was the overemphasis on amalgam fillings in our group, especially among clinical-year students. In contrast, the similar cohort investigated by Spaveras and Antoniadou [55] regarded amalgam as having a moderate burden on the environment. However, in the age group analysis of our cohort, we observed that as age increases, so does the level of understanding regarding dental procedures with the highest carbon footprint, an aspect highlighted by the limited or even absent knowledge among younger preclinical-year students on this topic.

Romanian dental students demonstrated significant awareness (38.8%) regarding the importance of reducing waste and using eco-friendly materials, within the responses to the tenth question. In contrast, one of the few studies focusing on dental students as a target population, conducted in Saudi Arabia, reported only limited student interest in sustainability policies and protocols embedded within their institutions, such as those addressing energy and waste management [69]. The penultimate insight into our students’ perspectives on sustainability issues in dentistry—addressed in the 11th question—explored how dental students perceive the need for university-led initiatives in this area. Understanding their evaluation of the university’s sustainability practices is particularly important, as it provides decision-makers with valuable insights into the institution’s performance from the viewpoint of one of its primary stakeholder groups. This need was echoed more than a decade ago by students at University Sains Malaysia, who sent a clear signal that sustainability should be approached holistically—addressing both on-campus and off-campus initiatives [70]. In line with this global sentiment, a significant proportion of dental students in Bucharest (62.7%) also expressed a strong interest in having the university implement sustainability initiatives, with female students demonstrating a particularly high level of support for such actions. This perspective confirms the study hypothesis, highlighting student awareness, gender disparities in engagement, and the crucial role of universities in sustainability.

The second objective of our study, which aimed to identify the need for integrating an elective course on sustainability into the dental curriculum among students, was reached through the responses to the 12th question, as expected, following the predominant result obtained previously. Female students again demonstrated greater awareness of the environmental impact of the dental profession due to their higher enthusiasm for future elective courses on sustainability, which could be interpreted outside the context of gender differences in motivations for additional courses. Despite arguments suggesting limited room for additional curricular content [54] and recommendations that embedding environmental sustainability in dental education does not necessarily require a complete overhaul of the curriculum, it remains imperative to emphasize the expressed needs of dental students. For instance, findings from a multi-center, nationally representative study conducted in the UK by Durnall et al. [58] revealed that an impressive 97.7% of dental students believed that introducing undergraduate-level education on environmental sustainability would positively impact their professional development, irrespective of its format. Similarly, recent reports from the UK, the United States [71], and Saudi Arabia [70] highlight waves of positive attitudes among dental students globally toward embedding sustainability within their undergraduate programs. Although these waves remain few, originating from different corners of the globe, they consist of the enthusiasm recorded in our research. In this context, 65% of respondents expressed a positive attitude toward an elective course on sustainability in dentistry if introduced at the University of Bucharest, supporting the second objective of our study. This strong majority aligns with findings from the 2014 Higher Education Academy and the National Union of Students study of 5000 students, which similarly reported that two-thirds of UK respondents supported integrating sustainability into university courses [72].

Empowered youth and a lifestyle oriented toward environmental consciousness hold immense potential as catalysts for long-term environmental protection and stewardship [73]. Scientific investigations have revealed that university students possess a certain level of awareness regarding energy usage and its associated carbon emissions [74]. In this regard, the thirteenth question of our survey highlighted that a significant majority of students (58.6%) expressed interest in adopting an environmentally linked lifestyle, which aligns with the growing awareness of the importance of sustainability in today’s global context. However, as the findings also revealed, there were notable gaps in the participants’ ability to effectively implement sustainability practices. This discrepancy points to the complexities of translating environmental awareness into practical, everyday actions. While students recognize the importance of sustainability, their lack of knowledge regarding specific, actionable practices could hinder their ability to engage in environmental initiatives in a meaningful way. These gaps may be attributed to the insufficient involvement of students in sustainability-focused organizations or initiatives, a deficiency observed in 85.3% of the cohort surveyed, as revealed by the penultimate question of our survey. The absence of direct engagement with sustainability-related groups, either within the university or in external contexts, seems to narrow their exposure to knowledge that could empower them to translate them into sustainable practices. It is crucial to note that mere awareness without practical knowledge can lead to knowing–doing gap [75], a phenomenon echoed also in previous research. Null et al. [76] found a similar pattern in their study of 291 students, where pro-environmental attitudes were present but students lacked actionable knowledge. Without proper exposure to sustainability learning, students may struggle to apply theoretical knowledge. Age group analysis in our study showed greater involvement in sustainability among older students, confirming the hypothesis that older students demonstrate greater sustainability knowledge and awareness.

Putting young people at the center of a circular economy responds to the pressing challenges of climate change and environmental degradation, the reuse, re-manufacturing, and recycling of products offering a promising path for sustainable economic growth. In a more hidden form, the fifteenth and final question aimed to assess the extent to which students actually practice the four principles of sustainability in their daily lives. Starting from the “reduce” principle, focused on reducing consumption (e.g., avoiding unnecessary actions and purchases), and proceeding to the “reuse” principle (e.g., reusing, donating, or altering unnecessary items that can have a “second life”), the “recycle” principle (e.g., recycling waste), and the “refuse” principle (e.g., refusing unnecessary purchases), the majority of our students regularly act according to three of these principles. Additionally, females, contrary to their purchasing profile [77], showed a greater awareness compared to male students regarding the intention to reduce their purchases, thus confirming the hypothesis regarding gender differences, through all the previously presented aspects, particularly in terms of awareness among female students about the environmental impact of traditional dentistry. However, they do not seem to be open to the concept of reusing goods. The specialized literature does not abound in data on promoting sustainability through the reuse principle of specific (dental-related) or non-specific goods within this population category. Moreover, there appears to be a notable gap or even ignorance regarding the various materials in dentistry that could potentially be reused. Examples include phosphate-bonded investments, dental gold, alginate, orthodontic brackets (ceramic and metallic), and dental alloys [78]. For this reason, Wang et al. have recommended further research to thoroughly understand undergraduates’ reuse intention in the context of higher education [79]. Additionally, variable predictors for the practice of reusing goods among students—especially in higher educational institutions—must be considered. These predictors include attitudes toward reuse, moral norms, and green university initiatives. 

As the assessment of dental students’ knowledge of sustainability came to an end, significant study limitations were identified. For instance, our cross-sectional study revealed awareness of environmentally sustainable dentistry practices among students. However, it could not determine whether this awareness was due to exposure to practical applications, parental education, social media, or other factors. Additionally, such a study could not assess how students’ knowledge and attitudes toward sustainability evolved over time with the inclusion of sustainability content in the dental curriculum. In terms of data generalizability, the study was conducted at a single university in Romania (Carol Davila University of Medicine and Pharmacy). Conducting this study within a single dental academic institution inherently limits the external validity of our findings. This constraint restricts our ability to generalize the results to other worldwide dental schools or student populations. The cultural context in which the study was carried out might have influenced the students’ attitudes toward sustainability. For example, students from other countries or regions might have had different levels of exposure to sustainability practices in their education or personal lives, making it difficult to generalize the findings to dental students at other institutions or in other countries.

One more methodological limitation was related to the response rate. The study achieved a 26.06% response rate (469 out of 1800 students), which was rather moderate. Although the calculated sample size (143 participants) was exceeded and provided sufficient statistical power, the low response rate could have introduced non-response bias. Students who chose not to participate may have held different views or behaviors regarding sustainability in dentistry, thus limiting the generalizability of the results to the entire student population.

Another limitation was the disproportionate representation of female students and those aged 18–24, which may have skewed the results, particularly regarding gender-based differences in sustainability perspectives and behaviors. For example, female students were significantly more likely to express interest in sustainability-related university initiatives and courses and to engage in sustainable behaviors such as recycling and purchasing organic food. As a result, the findings may not fully represent the broader student population, particularly male or older students who may have different attitudes and experiences related to sustainability. Additionally, the modest response rate among clinical students could further impact the representativeness of the results by introducing response bias.

Even so, to the best of our knowledge, this study is conducted with the largest number of responding students on the topic of sustainability in dental practice, carried out at a single dental school in Europe to date. A major future perspective would be the reform of Romania’s dental education system to include sustainability as a core competency. Given that sustainability is already being integrated into dental curricula in North America and Europe, Romanian dental schools could look to international institutions for guidance. Upcoming collaboration between Romanian faculties and international universities could facilitate knowledge exchange and provide models for integrating sustainability. Future curriculum development should integrate an elective course on sustainability, covering eco-friendly dental practices, sustainable materials, and environmental consciousness. Students would apply theoretical knowledge through actions such as selecting eco-friendly materials, using digital technologies to reduce carbon footprints, adopting energy-efficient practices, prioritizing minimally invasive techniques, and implementing sterilization methods that minimize waste.

In light of the growing global demand for environmentally responsible professionals, these efforts will ensure that dental students are well-prepared with the knowledge and skills necessary to meet the evolving expectations of patients, regulatory bodies, and society at large.

## 5. Conclusions

This study provides a significant step forward in understanding dental students’ knowledge and awareness of dental sustainability at Carol Davila University of Medicine and Pharmacy in Bucharest, a critical hub for academic innovation and leadership in Romania. The results revealed notable demographic trends, such as greater awareness and engagement among female students and preclinical-year cohorts, contrasted by gaps in knowledge among clinical-year students, particularly regarding the concept of sustainability, the environmental impact of dental practices, and dental procedures with the highest carbon footprint. These gaps are significant, considering that the most environmentally unsustainable dental specialties—such as prosthodontics, orthodontics, and oral surgery—are predominantly taught during the fifth and sixth years of study.

## Figures and Tables

**Table 1:** Distribution of students according to age group and other items answered in the survey.

Q9. Scale and Polish/Age Group	18–24 Years	25–30 Years	30+ Years	*p* *
Other answer	350 (79.9%)	16 (61.5%)	0 (0%)	<0.001
Affirmative	88 (20.1%)	10 (38.5%)	5 (100%)	
Q14. Initiative involvement	18–24 years	25–30 years	30+ years	*p* *
Negative	380 (86.8%)	17 (65.4%)	3 (60%)	0.005
Affirmative	58 (13.2%)	9 (34.6%)	2 (40%)	
Q15. Public transport	18–24 years	25–30 years	30+ years	*p* *
Other answer	197 (45%)	18 (69.2%)	4 (80%)	0.014
Affirmative	241 (55%)	8 (30.8%)	1 (20%)	

* Fisher’s Exact Test.

**Table 2:** Distribution of students according to gender and other items answered in the survey.

Q4. Sustainability Concept/Gender	Female	Male	*p* *
Ecological	102 (33.7%)	18 (16.1%)	0.001
Durability	138 (45.5%)	74 (66.1%)	
Economic	31 (10.2%)	9 (8%)	
Other	32 (10.6%)	11 (9.8%)	
Q6. Sustainability relevancy	Female	Male	*p* *
For environmental protection	95 (39.4%)	15 (17.9%)	0.001
For promoting the patient’s health	29 (12%)	9 (10.7%)	
For long-lasting procedures	17 (7.1%)	12 (14.3%)	
For promoting material quality	27 (11.2%)	8 (9.5%)	
For increasing the quality–price ratio	7 (2.9%)	2 (2.4%)	
For self-evolution in the dental practice	66 (27.4%)	38 (45.2%)	
Q7. Patient paperwork and records	Female	Male	*p* *
Other answer	208 (60.8%)	91 (71.7%)	0.031
Affirmative	134 (39.2%)	36 (28.3%)	
Q7. Dental office lighting and energy	Female	Male	*p* *
Other answer	250 (73.1%)	107 (84.3%)	0.014
Affirmative	92 (26.9%)	20 (15.7%)	
Q11. University—initiatives	Female	Male	*p* *
Yes	207 (66.3%)	59 (52.7%)	0.003
No	69 (22.1%)	44 (39.3%)	
Undecided	36 (11.5%)	9 (8%)	
Q12. Elective course—Sustainability	Female	Male	*p* *
Yes	242 (70.8%)	63 (49.6%)	<0.001
No	24 (7%)	18 (14.2%)	
Undecided	76 (22.2%)	46 (36.2%)	
Q15. Organic food	Female	Male	*p* *
Other answer	238 (69.6%)	101 (79.5%)	0.037
Affirmative	104 (30.4%)	26 (20.5%)	
Q15. Recycle waste	Female	Male	*p* *
Other answer	152 (44.4%)	71 (55.9%)	0.029
Affirmative	190 (55.6%)	56 (44.1%)	
Q15. Buy less	Female	Male	*p* *
Other answer	184 (53.8%)	89 (70.1%)	0.002
Affirmative	158 (46.2%)	38 (29.9%)	

* Fisher’s Exact Test.

**Table 3:** Distribution of students according to study year and other items answered in the survey.

Q4. Sustainability Concept/Study Year	I–III	IV–VI	*p* *
Ecological	84 (25.8%)	36 (40.4%)	0.022
Durability	169 (51.8%)	43 (48.3%)	
Economic	36 (11%)	4 (4.5%)	
Other	37 (11.3%)	6 (6.7%)	
Q5. Sustainability awareness importance—Score	I–III	IV–VI	*p* **
Average ± SD	8.61 ± 1.82	8.24 ± 1.92	0.028
Median (IQR)	9 (8–10)	9 (7–10)	
Q7. Patient paperwork and records	I–III	IV–VI	*p* *
Other answer	252 (67.2%)	47 (50%)	0.003
Affirmative	123 (32.8%)	47 (50%)	
Q9. Amalgam and composite fillings	I–III	IV–VI	*p* *
Other answer	196 (52.3%)	35 (37.2%)	0.011
Affirmative	179 (47.7%)	59 (62.8%)	
Q9. Endodontic treatment	I–III	IV–VI	*p* *
Other answer	303 (80.8%)	85 (90.4%)	0.032
Affirmative	72 (19.2%)	9 (9.6%)	
Q9. I don’t know	I–III	IV–VI	*p* *
Other answer	258 (68.8%)	75 (79.8%)	0.042
Affirmative	117 (31.2%)	19 (20.2%)	
Q14. Initiative involvement	I–III	IV–VI	*p* *
Negative	327 (87.2%)	73 (77.7%)	0.023
Affirmative	48 (12.8%)	21 (22.3%)	

* Fisher’s Exact Test, ** Mann–Whitney U Test.

## Data Availability

The data presented in this study are available from the corresponding authors upon request.

## Supplementary Materials

The following supporting information can be downloaded at: https://www.mdpi.com/article/10.3390/dj13030114/s1, Supplementary File Table S1. Knowledge and Awareness regarding Sustainability among Dental Students in Bucharest, Supplementary File Table S2 Socio-demographic characteristics of the analyzed students (Q1–Q3), Supplementary File Table S3 Distribution of the students according to the Type from survey answers (Q4–Q13).
