# Supplementary material for: Is Sustainability Part of the Drill? Examining Knowledge and Awareness Among Dental Students in Bucharest, Romania
Source: Dent J (Basel). 2025 Mar 5;13(3):114. doi: 10.3390/dj13030114 (PMC11941699; doi:10.3390/dj13030114)
Supplement: Supplementary file 1 [file dentistry-13-00114-s001.zip › dentistry-3462279-supplementary.pdf]

Table S1. Knowledge and Awareness regarding Sustainability among Dental Students in Bucharest

Question Number	Question Content	Question Type	Answer Options / Answer Coding
**Q1**	Which year/programme are you in?	Single Choice	• First<br>• Second<br>• Third<br>• Fourth<br>• Fifth<br>• Sixth
**Q2**	What best describes your gender?	Single Choice	• Male,<br>• Female<br>• Other<br>• Prefer not to say
**Q3**	What age are you?	Single Choice	• 18-24,<br>• 25-30<br>• 30
**Q4**	What does the term 'sustainability' mean to you? (Describe in one sentence)	Open-ended	•Ecological<br>•Durable<br>•Economic<br>•Other
**Q5**	How important is it for dentists to be aware of sustainability practices in their sector?	Likert Scale (1-10)	1 - Not important<br>5 - There are other areas dentists need to pay attention to<br>10 - Absolute priority
**Q6**	How do you think sustainability is relevant in the dental profession?	Open-ended	•For environmental protection;<br>•. For promoting the patient's health<br>•. For long-lasting procedures<br>•. For promoting material quality<br>•. For increasing the quality-price ratio<br>•. For self-evolution in dental practice
**Q7**	Which practices associated with dentistry have the highest adverse environmental impact, in your opinion?	Multiple Choice	• Use of specialist materials<br>• Dental equipment<br>• Patient care aspects, such as single use plastics and materials<br>• Patient paperwork & records<br>• Dental office lighting & energy<br>• Commute and use of transport to and from the dental office by the patients

**Q8**	Which one of the following activities do you consider to have the most effect on improving environmental impact?	Multiple Choice	• Use of modern dental materials<br>• Use of modern techniques and technologies (3D Printing, CAD/CAM, etc.)<br>• Digital data management & records • Reducing the use of plastic medical disposals<br>• Reducing the water and electricity consumption<br>• Educating staff and patients<br>• Other
**Q9**	What are the top 3 dental procedures with the largest carbon footprint?	Multiple Choice	• Scale and polish,<br>• Amalgam and composite fillings<br>• Acrylic dentures<br>• Radiographs<br>• Extractions<br>• Non-precious metal crowns<br>• Fluoride varnish<br>• Endodontic treatment<br>• Precious metal crowns<br>• Metal dentures<br>• Fissure sealants<br>• Porcelain crowns<br>• I don't know
**Q10**	What are the opportunities for the dental profession to improve its environmental impact?	Open-ended	• Dental office modernization<br>• Reducing waste and using eco-friendly materials<br>•Acquiring more durable materials<br>•Self-education about environmentally friendly practices
**Q11**	Are there any initiatives that you would like to see the university undertake?	Open-ended	•Yes<br>•No<br>•Undecided
**Q12**	If there was an optional course (elective) on sustainability in dentistry, would you be interested to attend?	Single Choice	• Yes<br>• No<br>• Undecided, I need more information
**Q13**	How would you describe your lifestyle?	Multiple Choice	• I am very aware of my environmental impact and it is a big part of all of my decisions<br>• I am very active in supporting social causes that are important to me<br>• I am worried about the environment and I have made some changes in my lifestyle however I probably could do more if I knew what else to do<br>• I am not doing anything about my environmental impact, but I would like some help to get started<br>• I am not concerned about my environmental impact

**Q14**	Are you currently involved in any sustainability initiatives or organisations (on campus or outside)?	Multiple Choice	• No, I am not involved in any<br>• Yes, I am very active - please provide more details
**Q15**	Please choose the choices you try to make regularly.	Multiple Choice	• Walk or cycle to work/university campus<br>• Use only public transport<br>• Turn off lights when not in use<br>• Use energy efficient light bulbs, • Buy organic food<br>• Recycle waste<br>• Buy less<br>• Buy used/ refurbished goods<br>• Other

Table S2. Socio-demographic characteristics of the analyzed students (Q1-Q3)

Parameter (N=469)	Value (Nr. %)
Study year	
First year (preclinical)	132 (28.1%)
Second year (preclinical)	135 (28.8%)
Third year (preclinical)	108 (23%)
Fourth year (clinical)	12 (2.6%)
Fifth year (clinical)	62 (13.2%)
Sixth year (clinical)	20 (4.3%)
Gender	
Female	342 (72.9%)
Male	127 (27.1%)
Age group	
18-24 years	438 (93.4%)
25-30 years	26 (5.5%)
30+ years	5 (1.1%)

Table S3. Distribution of the students according to the Type from survey answers (Q4-Q13).

Question	Answer (Nr., %)
**Q4.What does the term 'sustainability' mean to you? (N=415)**	
Ecological	120 (28.9%)
Durability	212 (51.1%)
Economic	40 (9.6%)
Other	43 (10.4%)
**Q5.How important is it for dentists to be aware of sustainability practices in their sector?**	
Average ± SD, Median (IQR)	8.54 ± 1.85, 9 (8-10)
**Q6.How do you think sustainability is relevant in the dental profession? (N=325)**	
For environmental protection	110 (33.8%)
For promoting the patient's health	38 (11.7%)
For long-lasting procedures	29 (8.9%)
For promoting material quality	35 (10.8%)
For increasing the quality-price ratio	9 (2.8%)
For self-evolution in the dental practice	104 (32%)
**Q7.Which activities associated with dentistry have the highest adverse environmental impact, in your opinion?**	
Use of specialist materials	217 (46.3%)
Dental equipment	205 (43.7%)
Patient care aspects such as single use plastics and materials	231 (49.3%)
Patient paperwork & records	170 (36.2%)
Dental office lighting & energy	112 (23.9%)
Commute and use of transport to and from the dental office by the patients	64 (13.6%)
**Q8.Which one of the following activities do you consider to have the most effect on improving environmental impact?**	
Use of modern dental materials	103 (22%)
Use of modern techniques and technologies	159 (33.9%)
Digital data management & records	59 (12.6%)
Reducing the use of plastic medical disposals	95 (20.3%)

Reducing the water and electricity consumption	13 (2.8%)
Educating staff and patients	38 (8.1%)
**Q9.What are the top 3 dental procedures with the largest carbon footprint?**	
Scale and polish	103 (22%)
Amalgam and composite fillings	238 (50.7%)
Acrylic dentures	105 (22.4%)
Radiographs	173 (36.9%)
Extractions	58 (12.4%)
Non-precious metal crowns	104 (22.2%)
Fluoride varnish	62 (13.2%)
Endodontic treatment	81 (17.3%)
Precious metal crowns	91 (19.4%)
Metal dentures	160 (34.1%)
Fissure sealants	30 (6.4%)
Porcelain crowns	66 (14.1%)
Examination	136 (29%)
**Q10.What are the opportunities for the dental profession to improve its environmental impact? (N=302)**	
Dental office modernization	89 (29.5%)
Reducing waste and using eco-friendly materials	116 (38.4%)
Getting more durable materials	24 (7.9%)
Self-education about environmentally friendly practice	**73 (24.2%)**
**Q11.Are there any initiatives that you would like to see the university undertake? (N=424)**	
Yes	266 (62.7%)
No	113 (26.7%)
Undecided	45 (10.6%)
**Q12.If there was an elective course on sustainability in dentistry, would you be interested to attend?**	
Yes	305 (65%)
No	42 (9%)
Undecided	122 (26%)

**Q13.How would you describe your lifestyle?**	
I am very aware of my environmental impact and it is a big part of all of my decisions	115 (24.5%)
I am very active in supporting social causes that are important to me	83 (17.7%)
I am worried about the environment and I have made some changes in my lifestyle, however I probably could do more if I knew what else to do	275 (58.6%)
I am not doing anything about my environmental impact, but I would like some help to get started	55 (11.7%)
I am not concerned about my environmental impact	18 (3.8%)
**Q14.Are you currently involved in any sustainability initiatives or organizations (on campus or outside)?**	
No, I am not involved in any	400 (85.3%)
Yes, I am very active	69 (14.7%)
**Q15. Please choose the choices you try to make regularly**	
Walk or cycle to work/university campus	205 (43.7%)
Use only public transport	250 (53.3%)
Turn off lights I'm not using them	327 (69.7%)
Use energy efficient light bulbs	185 (39.4%)
Buy organic food	130 (27.7%)
Recycle waste	246 (52.5%)
Buy less	196 (41.8%)
Buy used/ refurbished goods	67 (14.3%)
